# Fuzzy Adaptive Cubature Kalman Filter for Integrated Navigation Systems

**DOI:** 10.3390/s16081167

**Published:** 2016-07-26

**Authors:** Chien-Hao Tseng, Sheng-Fuu Lin, Dah-Jing Jwo

**Affiliations:** 1 Institute of Electrical Control Engineering, National Chiao Tung University, Hsinchu 300, Taiwan; chtseng.ece01g@g2.nctu.edu.tw (C.-H.T.); sflin@mail.nctu.edu.tw (S.-F.L.); 2 Department of Communications, Navigation and Control Engineering, National Taiwan Ocean University, Keelung 202, Taiwan

**Keywords:** integrated navigation, cubature Kalman filter, unscented Kalman filter, fuzzy logic

## Abstract

This paper presents a sensor fusion method based on the combination of cubature Kalman filter (CKF) and fuzzy logic adaptive system (FLAS) for the integrated navigation systems, such as the GPS/INS (Global Positioning System/inertial navigation system) integration. The third-degree spherical-radial cubature rule applied in the CKF has been employed to avoid the numerically instability in the system model. In processing navigation integration, the performance of nonlinear filter based estimation of the position and velocity states may severely degrade caused by modeling errors due to dynamics uncertainties of the vehicle. In order to resolve the shortcoming for selecting the process noise covariance through personal experience or numerical simulation, a scheme called the fuzzy adaptive cubature Kalman filter (FACKF) is presented by introducing the FLAS to adjust the weighting factor of the process noise covariance matrix. The FLAS is incorporated into the CKF framework as a mechanism for timely implementing the tuning of process noise covariance matrix based on the information of degree of divergence (DOD) parameter. The proposed FACKF algorithm shows promising accuracy improvement as compared to the extended Kalman filter (EKF), unscented Kalman filter (UKF), and CKF approaches.

## 1. Introduction

The Global Positioning System (GPS) and inertial navigation system (INS) have complementary operational characteristics and the synergy of both systems has been widely explored [1,2,3]. The GPS/INS integration is the adequate solution to provide a navigation system that has superior performance in comparison with either a GPS or an INS stand-alone system. The performance of GPS/INS integrated navigation system depends heavily on the design of sensor fusion, which is typically carried out through the extended Kalman filter (EKF) to estimate the state variables of a vehicle system and suppress the navigation measurement noise. Since most of the GPS/INS integration is based on the complimentary filter architecture, the feedback configuration leads to an EKF mode of operation in contrast to an ordinary linearized Kalman filter (LKF) that is associated with the feedforward case. Due to this reason, the system nonlinearity is remained for the feedback case as in the EKF. Although the Kalman filter has been shown to be a minimum mean square error (MMSE) estimator, the fact that EKF relies on the first order linearization of the system model to propagate the mean and covariance of the state might suffer from the performance degradation and divergence problem due to the linearization process and system miss-modeling. In addition, the complete calculation of the Jacobian matrix is cumbersome and time-consuming [4].

To better treat the nonlinearity, the unscented Kalman filter (UKF) [5,6,7] has been developed to address nonlinear state estimation in the context of control theory, which uses a finite number of sigma points to propagate the probability of state distribution through the nonlinear dynamics of system. Unlike the conventional EKF achieving first-order accuracy where the linearization process using the Jacobian matrices is involved, the UKF employs a minimal set of sigma points by deterministic sampling approach and at least the second order accuracy of the posterior mean and covariance can be captured. The UKF makes a Gaussian approximation with a limited number sigma points through the unscented transform (UT) [8,9]. When the sample points are propagated through the true nonlinear system, the posterior mean and covariance can be captured accurately to the second order of Taylor series expansion for any nonlinear system. However, the rounding errors of numerical calculation for UKF may destroy the non-negative and asymmetry of covariance matrix, therefore, the convergence rate of the UKF approach is slow and the system may also be unstable. Investigation of the nonlinear filtering approach to the integrated navigation problem has been seen using the sigma-point filters (SPFs) (e.g., [8,9]).

Under the Bayesian filtering framework, the CKF [10,11,12] provides a new direction to realize the nonlinear transformation. As opposed to the unscented transformation applied in UKF approach, the third-degree spherical-radial cubature rule is employed in the CKF to compute numerical integration encountered in the nonlinear Bayesian filter. The CKF can be treated as a special case of the UKF, which can be seen in some of the existing literature [13,14]. It is claimed in [14] that the UT is essentially a Gauss quadrature rule and other similar rules can also be applied. The spherical-radial cubature rule used in CKF is a special case of the quadrature rules. Although the positioning performance of the UKF and the CKF might be similar or even identical if the parameters of the UKF are well tuned, the possible negative weights of the center point in UT can be avoided through tuning the parameters [5]. Comparing with the UKF, there are no parameters to tune in CKF approximating nonlinear functions of the system and measurement. In general, using the additional tuning parameters of UKF compared to CKF is not a demerit. They provide flexibility. If one does not want to bother to tune them, just fix them as their default values, or just exclude the center point, and the CKF is automatically obtained.

The spherical-radial cubature rule is composed of two different integrals, spherical and radial integrals. This is based on the spherical-radial transformation and generates an even number of equally weighted cubature points. However, these integrals are then numerically computed by the spherical cubature rule and the Gaussian quadrature rule, respectively. The performance can be improved in terms of estimation accuracy, numerical stability and computational costs [11,12,13,14]. On the other hand, The UKF-calculated estimation covariance matrix is not always guaranteed to be positive definite, which it should be for correct functioning of the UKF. In view of the stability of the nonlinear filter, the CKF can effectively avoid round-off errors of numerical computation, and possesses more stable performance than the UKF and EKF [12]. The applicability and effectiveness of CKF based positioning system for sensor data fusion is presented in [15,16]. Liu et al. [16] presents an adaptive cubature Kalman filter (ACKF) algorithm based on maximum a posterior estimation and fading factor. In their research, the integration of inertial with distance measuring equipment (DME) was presented for the land-based navigation system. The adaptation of the measurement noise covariance matrix was introduced into the algorithm to enhance the applicability, where the adaptation module is based on the fading factor configuration with fuzzy logic.

There are basically two main factors that influence the estimation performance. First, there is model uncertainty because the adopted system model may not satisfy the actual state transition process. Second, there exist parameter uncertainties in the system model. The fixed value of covariance matrix cannot reflect the actual characteristics of the system error and measurement error. For a nonlinear system, the nonlinearity approximating capability may not be satisfied during the high-dynamic vehicle maneuvers, no matter what kind of filter: the Taylor series-based EKF, the unscented transformation-based UKF, or the spherical-radial transformation-based CKF is used. Using a nonlinear filter with fixed values of parameters in the varying dynamic environment has a risk on divergence due to modeling errors. To overcome the problem, an adaptive mechanism that dynamically identifies modeling errors can be adopted.

To deal with the system nonlinearity as well as the noise uncertainty, the fuzzy logic adaptive system (FLAS) is introduced [16,17,18,19] into the CKF to form the fuzzy adaptive cubature Kalman filter (FACKF), in which the FLAS is employed to continually adjust the noise strength in the internal model of the CKF framework, and tune the filter as well as possible. The FACKF scheme is applied to the GPS/INS navigation to improve the navigation estimation accuracy. In FACKF, the CKF is involved in the algorithm to estimate the nonlinear system states, while the process noise covariance is adjusted through the FLAS. The fuzzy logic reasoning system [19] based on the Takagi-Sugeno (T-S) model [20] is used in the FLAS. The fuzzy inference system (FIS) is constructed for obtaining the suitable weighting factor according to the time-varying change in dynamics. In addition, degree of divergence (DOD) parameters [21] are employed to identify the degree of change in vehicle dynamics. By monitoring the innovation information, the FLAS, as the filter’s internal module, is employed for dynamically adjusting the weighting factor based on the fuzzy rules so as to enhance the estimation performance and tracking capability.

The remainder of this paper is organized as follows. In Section 2, the preliminary background on the nonlinear filter is introduced, where the UKF and the CKF will be reviewed. The proposed sensor fusion strategy, FACKF approach, is introduced in Section 3. In Section 4, two illustrative examples are provided to evaluate the sensor fusion performance of the integrated navigation systems for the FACKF approach in comparison to those by conventional approaches. Conclusions are given in Section 5.

## 2. The Nonlinear Filters

Kalman filtering has been recognized as one of the most powerful state estimation techniques. The purpose of the Kalman filter is to provide the estimation with minimum error variance. The UKF is a nonlinear version of the Kalman filter and is widely used for the navigation sensor fusion. The unscented Kalman filtering deals with the case governed by the nonlinear stochastic difference equations:

(1) $$x_{k+1}=f(x_{k},k)+w_{k}$$

(2) $$z_{k}=h(x_{k},k)+v_{k}$$

where the state vector is $x_{k}\in {ℜ}^{n}$, process noise vector is $w_{k}\in {ℜ}^{n}$, measurement vector is $z_{k}\in {ℜ}^{m}$, and measurement noise vector is $v_{k}\in {ℜ}^{m}$. The vectors $w_{k}$ and $v_{k}$ are zero mean Gaussian white sequences having zero cross-correlation with each other:

(3) $$\begin{array}{c} E[w_{k}w_{i}^{T}]=\{\begin{array}{ll} Q_{k}, & i=k\\ 0, & i\neq k \end{array};\;E[v_{k}v_{i}^{T}]=\{\begin{array}{ll} R_{k}, & i=k\\ 0, & i\neq k \end{array};\;\\ \begin{array}{ccc} E[w_{k}v_{i}^{T}]=0 & & for\;all\;i\;and\;k \end{array} \end{array}$$

where $Q_{k}$ is the process noise covariance matrix, and $R_{k}$ is the measurement noise covariance matrix.

### 2.1. The Unscented Kalman Filter

The UKF was first proposed by Julier et al. [7] to address nonlinear state estimation in the context of control theory. The first step in the UKF is to generate the sigma points through the UT. One of the popular UT approaches is the scaled unscented transformation. Consider an n dimensional random variable x, having the mean $\hat{x}$ and covariance P, and suppose that it propagates through an arbitrary nonlinear function f. The unscented transform creates 2n+1 sigma vectors X (a capital letter) and weighted points W, given by

(4) $$X_{\left(0\right)}=\hat{x};\;X_{\left(i\right)}=\hat{x}+{\left(\sqrt{(n+\lambda )P}\right)}_{i}^{T};\;X_{\left(i+n\right)}=\hat{x}-{\left(\sqrt{(n+\lambda )P}\right)}_{i}^{T},\;i=1,\ldots ,n$$

(5) $$W_{0}^{\left(m\right)}=\frac{\lambda }{\left(n+\lambda \right)};\;W_{0}^{\left(c\right)}=W_{0}^{\left(m\right)}+(1-\alpha^{2}+\beta );\;W_{i}^{\left(m\right)}=W_{i}^{\left(c\right)}=\frac{1}{2(n+\lambda )},\;i=1,\ldots ,2n$$

where ${\left(\sqrt{(n+\lambda )P}\right)}_{i}$ is the *i*th row of the matrix square root. $\sqrt{(n+\lambda )P}$ can be obtained from the lower-triangular matrix of the Cholesky factorization; $\lambda =\alpha^{2}(n+\kappa )-n$ is a scaling parameter; α determines the spread of the sigma points around $\overset{⌢}{x}$; κ is a secondly scaling parameter; β is used to incorporate prior knowledge of the distribution of $\bar{x}$; $W_{i}^{\left(m\right)}$ is the weight for the mean associated with the *i*th point; and $W_{i}^{\left(c\right)}$ is the weight for the covariance associated with the *i*th point.

The sigma vectors are propagated through the nonlinear function to yield a set of transformed sigma points,

(6) $$y_{i}=f(X_{i}),\;i=0,\ldots ,2n$$

The mean and covariance of $y_{i}$ are approximated by a weighted average mean and covariance of the transformed sigma points as follows:

(7) $${\bar{y}}_{u}=\sum_{i=0}^{2n}W_{i}^{\left(m\right)}y_{i}$$

(8) $$P_{u}=\sum_{i=0}^{2n}W_{i}^{\left(c\right)}(y_{i}-{\bar{y}}_{u}){\left(y_{i}-{\bar{y}}_{u}\right)}^{T}$$

As compared to the EKF’s linear approximation, the unscented transformation is accurate to the second order for any nonlinear function. Table 1 shows the algorithm for implementation of the UKF, given as in Equations (9)–(18).

### 2.2. The Cubature Kalman Filter

A nonlinear Bayesian filter called the cubature Kalman filter (CKF) was presented by Arasaratnam and Haykin [9]. Proposed for nonlinear state estimation, the CKF is a Gaussian approximation of a Bayesian filter that provides a more accurate filtering estimate then existing Gaussian filters.

The Bayesian filter solution reduces to computing multi-dimensional integrals, whose integrands are of the form *nonlinear function* × *Gaussian density*. The CKF utilizes the property of the efficient numerical integration method known as spherical-radial cubature rule for those multi-dimensional integrals [22]. Based on the third-degree cubature rule, a set of 2n points are selected in the CKF to capture the mean and covariance in each update cycle. The cubature rule to approximate an n-dimensional Gaussian weighted integral is

(19) $$\int_{R^{n}}f(x)Ν(x;m_{x},P)dx\approx \frac{1}{2n}\sum_{i=1}^{2n}f(m_{x}+\sqrt{P}\zeta_{i})$$

where f(x) is the arbitrary function, $R^{n}$ is domain of integration. $m_{x}$ is the mean of state x. $\sqrt{P}$ is a square root factor of the covariance matrix P satisfying the relation $P=\sqrt{P}{\sqrt{P}}^{T}$ and the set of 2n cubature point. $\xi_{i}$ is the *i*th cubature point.

The CKF algorithm involves the following stages: Firstly, it approximates the mean and variance of the probability distribution through a set of 2n cubature points with the same weight, propagates the cubature points through the nonlinear functions, and then calculates the mean and variance of the current approximate Gaussian distribution by the propagated cubature points. A set of 2n cubature points is given by $\left[\xi_{i},\omega_{i}\right]$, where $\xi_{i}$ is the *i*th cubature point and $\omega_{i}$ is the corresponding weight:

(20) $$\xi_{i}=\{\begin{array}{lll} \sqrt{n}{\left[1\right]}_{i}, & & i=1,2,\ldots ,n\\ -\sqrt{n}{\left[1\right]}_{i-n}, & & i=n+1,n+2,\ldots ,2n \end{array}$$

(21) $$\omega_{i}=\frac{1}{2n},\;i=1,2,\ldots ,2n$$

where ${\left[1\right]}_{i}\in {ℜ}^{n}$ denotes the *i*th column vector of the identity matrix $I_{n\times n}$.

Under the assumption that the posterior density at time k−1 is known, the steps involved in the time and measurement updates of the CKF are derived, given by Equations (22)–(35), summarized in Table 2. The kernel method of the CKF is that the mean and variance of probability distribution can be approximated by cubature points without any linearization of the system model. Thus, the CKF algorithm does not demand to calculate Jacobian matrices so that the truncation errors can be avoided.

Like the UKF, the CKF is another type of nonlinear filtering approach without linearization of nonlinear model. The CKF and UKF are distinguished in several aspects: (1) there has been emphasis on spherical-radial integration rule of the CKF approach which has desirable numerical accuracy criterion than on the efficiency; (2) the approaches to perform the Cholesky factorization on the error covariance matrix as the first step of both the time and measurement updates in each time step; (3) the CKF follows directly from the spherical-radial cubature rule for numerically computing Gaussian-weighted integrals whose important property is that it does not entail any free parameters, whereas the UKF introduces a nonzero scaling parameter; and (4) the covariance matrix in the UKF is not always guaranteed to be positive definite, hence, the decomposition of the covariance matrix is unavailable. In view of numerical stability, the use of UT in the design of the UKF may marginally improve its performance at expense of a reduced numerical stability because the estimation error covariance matrix is not always guaranteed to be positive semidefinite. Compared to the CKF, which builds on the numerical-integration perspective of Gaussian filters, employs a third-degree spherical-radical cubature rule to compute Gaussian-weighted integrals numerically and is a relatively derivative-free nonlinear filter with improved performance over the UKF in terms of numerical stability. In addition, they are fundamentally different in sampling point set: for the sigma-point set, the stem at the center is highly significant as it carries more weight, whereas the cubature point set does not have a stem at the center and thus does not have the numerical instability problem of UKF. To avoid the numerical instability problem, the CKF can effectively improve filtering stability [11,12,13,14].

## 3. The FLAS-assisted CKF Strategy

The fuzzy logic adaptive system is introduced to the CKF framework to enhance improvement for vehicular navigation in high-dynamic environments. There are many state estimation methods that were found to have practical applications for vehicle positioning and navigation in various dynamic environments, with a pursuit of estimation accuracy and adaptive capability. Although the cubature-based CKF solution solves the nonlinear approximation issue in a different way from UKF, it still has to meet the requirements of robust estimation and performance stability in high dynamic environments. In the design of CKF, tolerance to the uncertainty factors, including the noise uncertainty and system modeling inaccuracy, is not a high concern, which require the development of robust demand for CKF filtering scheme. The problem in this work is described as a strategy to adaptively adjust the weighting factor in CKF framework and achieve a balance between robustness and estimation performance.

### 3.1. The Fuzzy Logic Adaptive System (FLAS)

**Figure 1:**
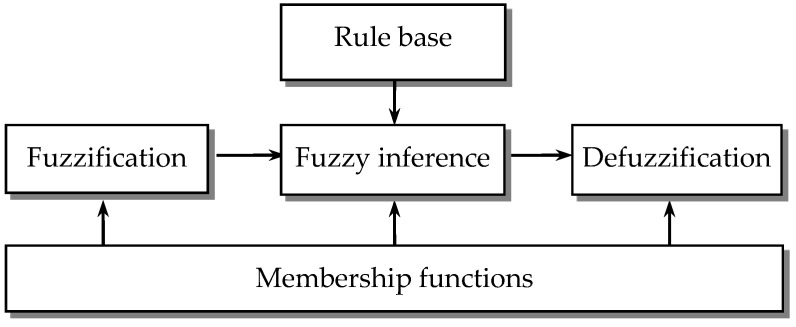
A fuzzy system.

Fuzzy modeling is the method of describing the characteristics of a system using fuzzy rules, which are linguistic IF-THEN statements involving fuzzy sets, fuzzy logic, and fuzzy inference. There are two major types of fuzzy rules exist, namely, Mamdani fuzzy rules and Takagi-Sugeno (T-S) fuzzy rules. The designed FLAS utilizes a fuzzy inference system of Takagi-Sugeno type, which has special properties since it represents the nonlinear systems in the form of an interpolation between local linear models. A typical fuzzy system consists of three components: fuzzification, fuzzy reasoning (fuzzy inference), and fuzzy defuzzification, as shown in Figure 1. The fuzzification process converts a crisp input value to a fuzzy value, the fuzzy inference is responsible for drawing calculations from the knowledge base, and the fuzzy defuzzification process converts the fuzzy actions into a crisp action.

The T-S fuzzy model represents the conclusion by functions. A typical rule in the T-S fuzzy model has the form:

Model Rule i: IF Input $x_{1}$ is $F_{1}^{1}$ and Input $x_{2}$ is $F_{2}^{1}$ and Input $x_{n}$ is $F_{n}^{1}$
THEN Output $y_{k}=f_{k}(x_{1},x_{2},\ldots ,x_{n})=C_{k0}+C_{k1}x_{1}+\cdots +C_{kn}x_{n}$.

where F is a triangle-shaped membership function of the input variable vector, $C_{ki}(i=0\sim n)$ are constants in the *k*-th rule. For a zero-order model, the output level is a constant:

Model Rule i: IF Input $x_{1}$ is $F_{1}^{1}$ and Input $x_{2}$ is $F_{2}^{1}$; THEN Output $y_{k}=C_{10}$.

For the first-order model, the fuzzy rule can be expressed in the form:

Model Rule i: IF Input $x_{1}$ is $F_{1}^{1}$ and Input $x_{2}$ is $F_{2}^{1}$
THEN Output $y_{k}=C_{10}+C_{11}x_{1}+C_{12}x_{2}$.

where $F_{1}^{1}$ and $F_{2}^{1}$ are fuzzy sets and $C_{10}$, $C_{11}$ and $C_{12}$ are constants.

In the FLAS, the weighted average method of defuzzification to find the crisp output. The weighted average defuzzification method can be expressed as:

(36) $$y=\sum_{k=1}^{M}w_{k}.y_{k}$$

where the weights $w_{k}$ are computed as:

(37) $$w_{k}=\frac{\prod_{i=1}^{n}\mu_{F_{i}^{k}}(x_{i})}{\sum_{j=1}^{M}\left[\prod_{i=1}^{n}\mu_{F_{i}^{j}}(x_{i})\right]}$$

with $\sum_{i=1}^{M}w_{i}=1$, and the μ’s represent the membership function.

### 3.2. FLAS-Assisted CKF for Vehicle Navigation

In processing navigation states using the model-based filters as discussed in this paper, the time varying parameters are considered uncertainties to exist in the covariance matrices. The FLAS module in the CKF is employed to adapt the filter on-line. The T-S fuzzy model was used to directly estimate the variance and covariance components for the measurements and adapt the CKF. Generally, when the covariance is becoming large and is deviating from zero mean, the filter will be toward to the instability. To avoid filter divergence and improve the robustness, the fuzzy logic system is used to adapt the CKF by selecting the appropriate weighting factor ε, which is used to adaptively adjust the process noise covariance based on a degree of divergence (DOD) parameters.

The innovation information is a critical factor for the filter, including the CKF. For the filter to be optimal, the innovation is a zero-mean Gaussian white noise. Therefore, the performance of the CKF can be monitored using the value of the innovation information. It is defined as the discrepancy between actual measurements and predicted measurements:

(38) $$\upsilon_{i}=z_{k}-{\hat{z}}_{k|k-1}$$

The DOD parameters for identifying the degree of change in vehicle dynamics need to be defined. Examples of possible approaches are given as follows. The innovation information at the present epoch is employed for timely reflect the change in vehicle dynamics. The DOD parameter $\mu_{1}$ defined as the averaged magnitude of innovation at the present epoch can be employed for timely reflection of the time-varying vehicle dynamics:

(39) $$\mu_{1}=\frac{1}{m}\sum_{i=1}^{m}\left|\upsilon_{i}\right|$$

where $\upsilon_{i}={\left[\upsilon_{1}\upsilon_{2}\ldots \upsilon_{m}\right]}^{T}$, and m is the number of measurements (e.g., number of satellites in the tightly-coupled configuration). Furthermore, the trace of innovation covariance matrix at present epoch divided by the number of measurements employed for navigation processing can also be used:

(40) $$\mu_{2}=\frac{\upsilon_{i}^{T}\upsilon_{i}}{m}$$

In the FLAS, the DOD parameters are employed as the inputs for the fuzzy inference engines. By monitoring the DOD parameters, the FLAS is able to on-line tune the weighting factor according to the innovation information. In this work, the fuzzy logic used to perform adaptation involves two parameters, $\mu_{1}$ and $\mu_{2}$ [21]. For this reason, this scheme can adjust the process noise covariance adaptively and therefore improves estimation performance. The adjustments are performed simply introducing a weighting factor ε as the scaling factor of the process noise covariance, in the following way:

(41) $$Q_{k}\to \varepsilon \cdot Q_{k}$$

The FLAS is used to identify the appropriate weighting factor so as to keep the innovation sequence toward to the zero-mean white sequence. Two DOD parameters based on the innovation are chosen as the input variables for timely reflection of the time-varying vehicle dynamics. The output is the weighting factor for tuning the process noise covariance matrix. Generally, when the covariance is becoming large and is deviating from zero mean, the filter will be toward to the instability. In such cases, the process noise covariance needs to be increased by applying a larger weighting factor for compensating the modeling error. When the innovation mean and covariance are small, the residual between actual and predicted measurements are small as well, meaning that the two measurements match adequately well.

**Figure 2:**
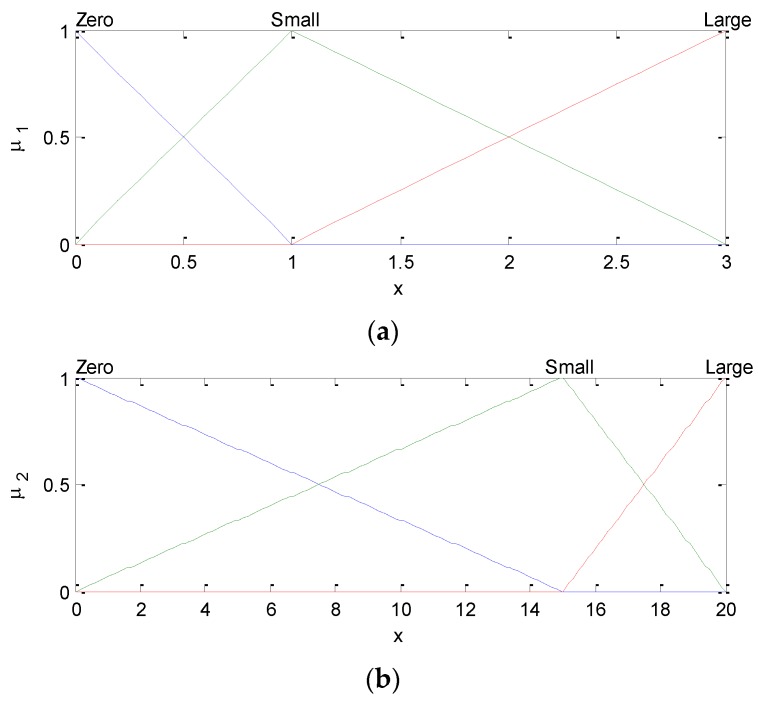
Membership functions of input fuzzy variables $\mu_{1}$ (**a**) and $\mu_{2}$ (**b**).

In this paper, the first-order T-S fuzzy model has been employed. The membership functions (MFs) of input fuzzy variables as shown in Figure 2, where the triangle MFs are involved. The presented FLAS has the IF–THEN form and consists of 9 rules:

1. IF $\mu_{1}$ is zero and $\mu_{2}$ is zero THEN ε is $C_{10}$
2. IF $\mu_{1}$ is zero and $\mu_{2}$ is small THEN ε is $C_{20}$
3. IF $\mu_{1}$ is zero and $\mu_{2}$ is large THEN ε is $C_{30}$
4. IF $\mu_{1}$ is small and $\mu_{2}$ is zero THEN ε is $C_{40}+C_{41}\mu_{1}+C_{42}\mu_{2}$
5. IF $\mu_{1}$ is small and $\mu_{2}$ is small THEN ε is $C_{50}+C_{51}\mu_{1}+C_{52}\mu_{2}$
6. IF $\mu_{1}$ is small and $\mu_{2}$ is large THEN ε is $C_{60}+C_{61}\mu_{1}+C_{62}\mu_{2}$
7. IF $\mu_{1}$ is large and $\mu_{2}$ is zero THEN ε is $C_{70}+C_{71}\mu_{1}+C_{72}\mu_{2}$
8. IF $\mu_{1}$ is large and $\mu_{2}$ is small THEN ε is $C_{80}+C_{81}\mu_{1}+C_{82}\mu_{2}$
9. IF $\mu_{1}$ is large and $\mu_{2}$ is large THEN ε is $C_{90}+C_{91}\mu_{1}+C_{92}\mu_{2}$

where $C_{ki}(i=0\sim 2)$ are constants in the *k*-th rule. Parameters $C_{ki}$ can be tuned for different rules based on a value calculated by the combination of the DOD parameters, $\mu_{1}$ and $\mu_{2}$. The parameter values selected in this work are as follows: $C_{10}=C_{20}=C_{30}=5$; $C_{40}=C_{50}=C_{60}=10$; $C_{70}=C_{80}=C_{90}=20$; $C_{41}=C_{51}=C_{61}=5$; $C_{71}=C_{81}=C_{91}=10$; $C_{42}=C_{52}=C_{62}=C_{72}=C_{82}=C_{92}=20$.

**Figure 3:**
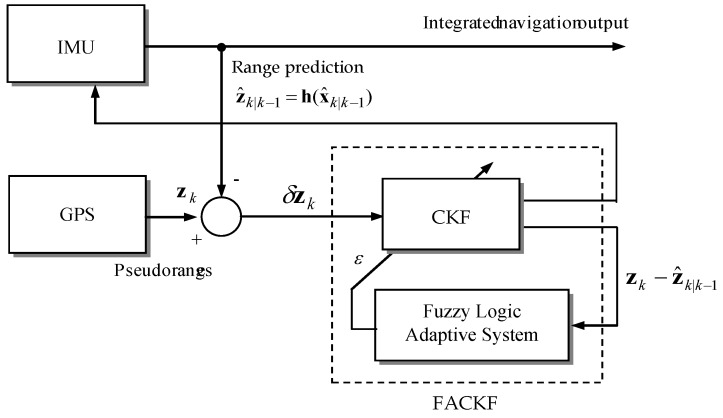
Architecture of the tightly-coupled GPS/INS (Global Positioning System/inertial navigation system) integrated navigation using the FACKF (fuzzy adaptive cubature Kalman filter)-feedback configuration.

Figure 3 shows the sensor fusion architecture of the tightly-coupled GPS/INS navigation based on the FLAS-assisted CKF filtering mechanism. In this research, the error state integrated navigation model with feedback configuration is used. The residual between GPS pseudorange and INS predicted range is used as the measurement of the CKF in the tightly-coupled configuration. For the loosely-coupled case, the measurement then is the residual of the position/velocity instead of pseudoranges/pseudorange rates. The block indicated by the dashed-line on the right-hand side is the fuzzy logic adaptive system (FLAS), which determines the value of weighting factor ε.

## 4. Results and Discussion

Numerical simulations have been carried out to evaluate the performance of the FACKF approach in comparison with those of EKF, UKF and CKF approaches for the integrated navigation data fusion. The commercial software Satellite Navigation (SATNAV) Toolbox by GPSoft LLC was employed for generating the satellite positions and pseudoranges after the test trajectory has been defined. It is assumed that the differential GPS mode is used and only the multipath and receiver thermal noise are included.

Assume that the differential GPS (DGPS) mode is used and most of the errors can be corrected, but the multipath and receiver thermal noise cannot be eliminated. The measurement noise variances $r_{pi}$ are assumed a priori known, which is set as 9 m^2^. Let each of the white-noise spectral amplitudes that drive the random walk position states be $S_{p}=0.003\left({m/s}^{2}\right)/\text{rad}/s$. Furthermore, let the clock model spectral amplitudes be $S_{f}=0.4\left(10^{-18}\right)s$ and $S_{g}=1.58\left(10^{-18}\right)s^{-1}$. In this paper, two illustrative examples are given to confirm the effectiveness of the FACKF approach by valuation of the performance for the various approaches including EKF, UKF, CKF, and FACKF. The simulation tests involve the scenarios of two-dimensional loose integration and three-dimensional tight integration. For both examples, several sets of parameters have been tested and two sets of parameters for the UKF are presented. The UKF parameters are set as α=2.5, β=2, κ=0.

### 4.1. Scenario 1 (Example for 2D Land Vehicle Navigation)

**Figure 4:**
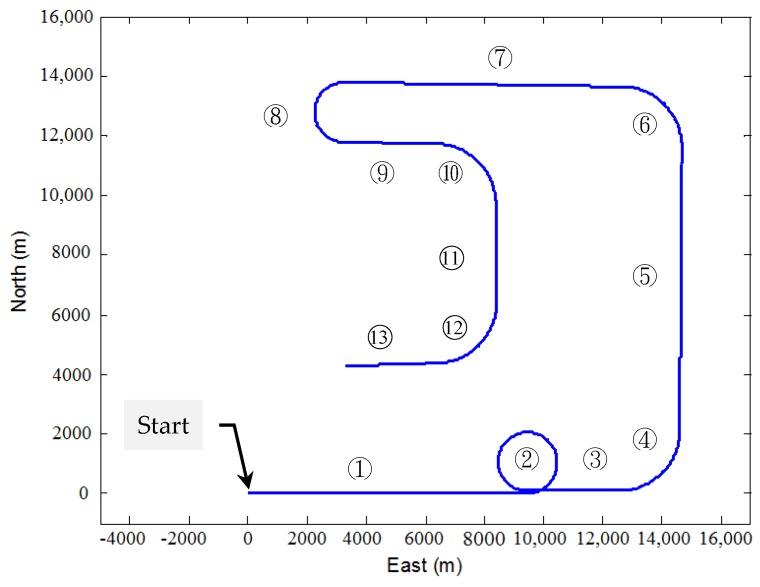
Two-dimensional vehicle trajectory for Scenario 1.

**Figure 5:**
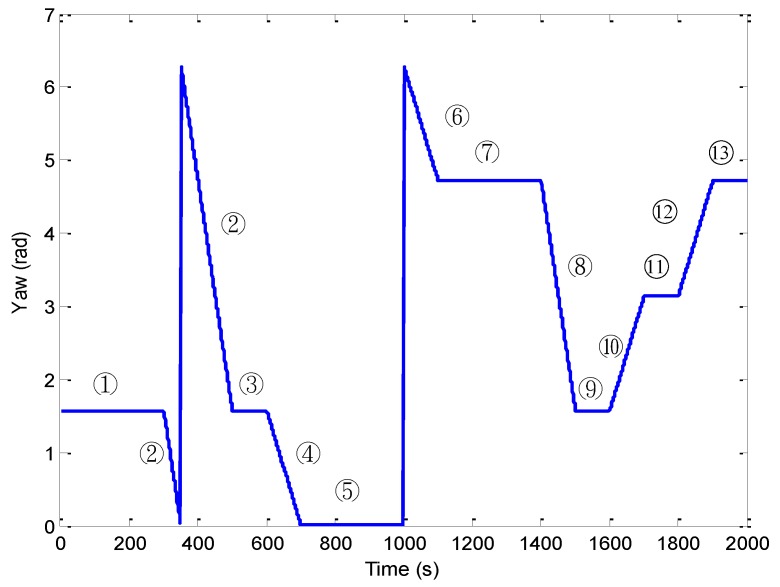
The yaw angle of the vehicle for two-dimensional simulation.

For the first test, a simulated vehicle originates from the (0, 0) m location in the ENU coordinate frame. A loosely-coupled GPS/INS integration configuration is used for this two dimensional case. The trajectory can be divided mainly into thirteen time intervals (or segments) according the dynamic characteristics, as indicated in Figure 4. Figure 5 shows the yaw angle of the vehicle for two-dimensional simulation. The vehicle is simulated to conduct constant-velocity, straight-line moving during seven time intervals, 0–300, 501–600, 701–1000, 1101–1400, 1501–1600, 1701–1800 and 1901–2000 s, all at a speed of 10π m/s. Furthermore, the higher dynamic maneuvering conducted counter-clockwise circular and turn motion during 301–500, 601–700, 1001–1100, 1401–1500, 1601–1700 and 1801–1900 s. Table 3 presents description of the vehicle motion for providing better insight into vehicle dynamic information in each time interval. The standard deviations of inertial sensors are $9\times 10^{-4}$ m/s^2^ for the accelerometers and $9\times 10^{-4}$ rad/s for the gyroscope, respectively.

The differential equations describing the two-dimensional inertial navigation state, where two accelerometers and one gyroscope are involved as follows:

(42) $$\left[\begin{array}{c} \dot{n}\\ \dot{e}\\ {\dot{v}}_{n}\\ {\dot{v}}_{e}\\ \dot{\psi } \end{array}\right]=\left[\begin{array}{c} v_{n}\\ v_{e}\\ a_{n}\\ a_{e}\\ \omega_{r} \end{array}\right]=\left[\begin{array}{c} v_{n}\\ v_{e}\\ \cos(\psi )a_{u}-\sin(\psi )a_{v}\\ \sin(\psi )a_{u}+\cos(\psi )a_{v}\\ \omega_{r} \end{array}\right]$$

where $\left[a_{u},a_{v}\right]$ are the measured acceleration in the body frame, and $\omega_{r}$ is the measured yaw rate in the body frame. The error model for INS is constructed by the navigation states augmented by the accelerometer biases and gyroscope drift:

(43) $$\frac{d}{dt}\left[\begin{array}{c} \delta n\\ \delta e\\ \delta v_{n}\\ \delta v_{e}\\ \delta \psi \\ \delta a_{u}\\ \delta a_{v}\\ \delta \omega_{r} \end{array}\right]=\left[\begin{array}{cccccccc} 0 & 0 & 1 & 0 & 0 & 0 & 0 & 0\\ 0 & 0 & 0 & 1 & 0 & 0 & 0 & 0\\ 0 & 0 & 0 & 0 & -a_{e} & \cos(\psi ) & -\sin(\psi ) & 0\\ 0 & 0 & 0 & 0 & -a_{n} & \sin(\psi ) & \cos(\psi ) & 0\\ 0 & 0 & 0 & 0 & 0 & 0 & 0 & 1\\ 0 & 0 & 0 & 0 & 0 & 0 & 0 & 0\\ 0 & 0 & 0 & 0 & 0 & 0 & 0 & 0\\ 0 & 0 & 0 & 0 & 0 & 0 & 0 & 0 \end{array}\right]\left[\begin{array}{c} \delta n\\ \delta e\\ \delta v_{n}\\ \delta v_{e}\\ \delta \psi \\ \delta a_{u}\\ \delta a_{v}\\ \delta \omega_{r} \end{array}\right]+\left[\begin{array}{c} 0\\ 0\\ u_{acc}\\ u_{acc}\\ u_{gyro}\\ u_{acc}^{b}\\ u_{acc}^{b}\\ u_{gyro}^{b} \end{array}\right]$$

which is utilized in the integration navigation filter as the inertial error model. In Equation (43), δn and δe represent the east and north position errors, respectively; $\delta v_{n}$ and $\delta v_{e}$ denote the east and north velocity errors, respectively; δψ indicate yaw angle; and $\delta a_{u}$, $\delta a_{v}$ and $\delta \omega_{r}$ are the accelerometer biases and gyroscope drift, respectively. The measurement model is written as

(44) $$\left[\begin{array}{c} \Delta n\\ \Delta e \end{array}\right]=\left[\begin{array}{c} n_{INS}\\ e_{INS} \end{array}\right]-\left[\begin{array}{c} n_{GPS}\\ e_{GPS} \end{array}\right]=\left[\begin{array}{cccccccc} 1 & 0 & 0 & 0 & 0 & 0 & 0 & 0\\ 0 & 1 & 0 & 0 & 0 & 0 & 0 & 0 \end{array}\right]\left[\begin{array}{c} \delta n\\ \delta e\\ \delta v_{n}\\ \delta v_{e}\\ \delta \psi \\ \delta a_{u}\\ \delta a_{v}\\ \delta \omega_{r} \end{array}\right]+\left[\begin{array}{c} v_{n}\\ v_{e} \end{array}\right]$$

**Figure 6:**
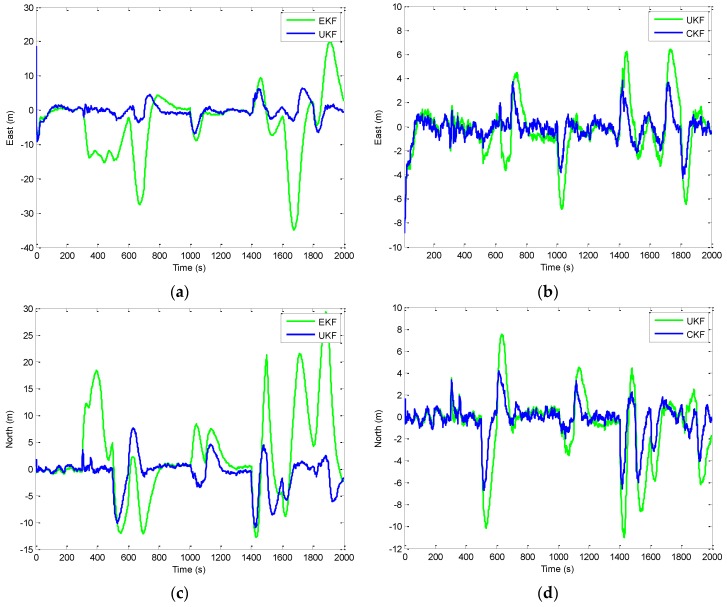
Position errors for EKF, UKF, and CKF—Scenario 1: (**a,c**) EKF versus UKF; and (**b,d**) UKF versus CKF.

**Figure 7:**
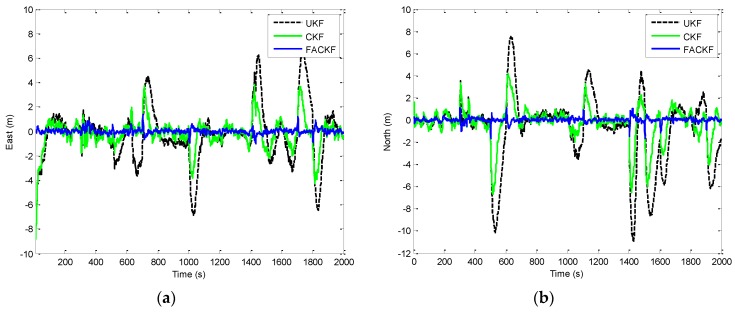
Comparison of position errors based on the UKF, CKF, and FACKF—Scenario 1. (**a**) East position error; (**b**) North position error.

**Figure 8:**
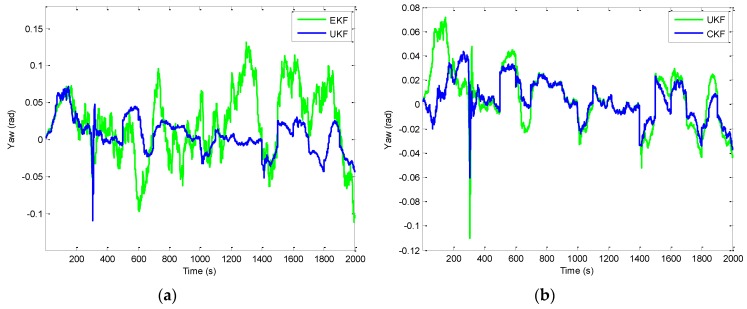
Comparison of yaw angle errors for EKF, UKF, and CKF—Scenario 1: (**a**) EKF versus UKF; and (**b**) UKF versus CKF.

**Figure 9:**
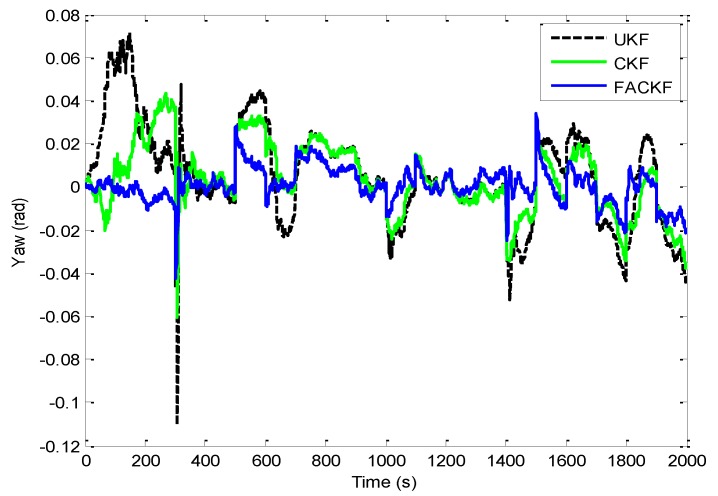
Comparison of yaw angle errors for UKF, CKF, and FACKF—Scenario 1.

Figure 6 shows the position errors based on the three filters: EKF, UKF, and CKF. The results for EKF versus UKF are presented by the two plots shown in the left column while those for UKF versus CKF in the right column. Figure 7 provides the comparison of position errors for the UKF, CKF and FACKF. In additions, the estimation performance for the Euler angles among various approaches is presented. The results for the yaw angle errors based on the EKF, UKF, and CKF are shown in Figure 8 and Figure 9. In Figure 8, the result for EKF versus UKF is shown in the left plot while that for UKF versus CKF in the right plot. Comparison of yaw angle errors for UKF, CKF, and FACKF is presented in Figure 9. The FACKF has demonstrated performance improvement capability when compared to the CKF and UKF, due to better treatment on nonlinearity caused by vehicle maneuvers. The FLAS is adopted to on-line determine the weighting factor in the FACKF, hence prevents the divergence problem, and results in improved navigation accuracy. Table 4 provides the summary of root mean square (RMS) errors and the time consumption for all the four approaches: EKF, UKF, CKF and FACKF. It can be seen that the incorporation of the FLAS mechanism can remarkably improve the estimation accuracy of the navigation states. Among the four approaches, the FLAS-assisted CKF strategy demonstrates superior navigation accuracy performance as compared to the other three approaches.

### 4.2. Scenario 2 (Example for 3D Navigation Environment)

The second example for the three-dimensional navigation case is presented for further confirmation of the robustness and effectiveness of the proposed method. The data for INS error specifications are taken from Crista IMU specifications [23], as shown in Table 5. The GPS data were generated at 1 Hz and the IMU has a data rate of 10 Hz.

**Figure 10:**
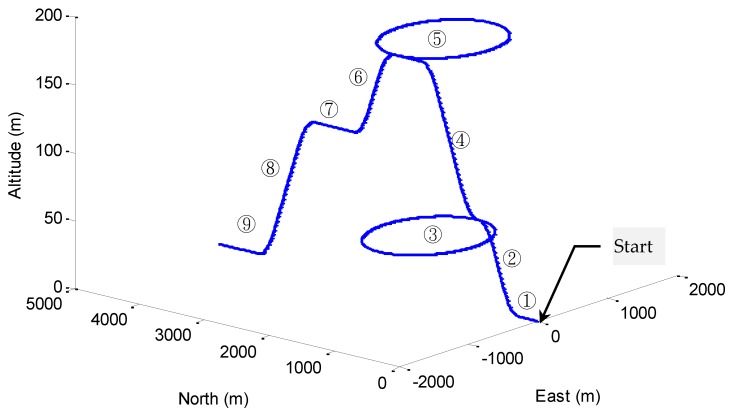
Three-dimensional vehicle trajectory for Scenario 2.

**Figure 11:**
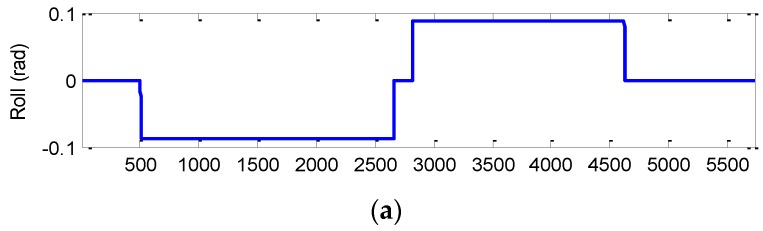

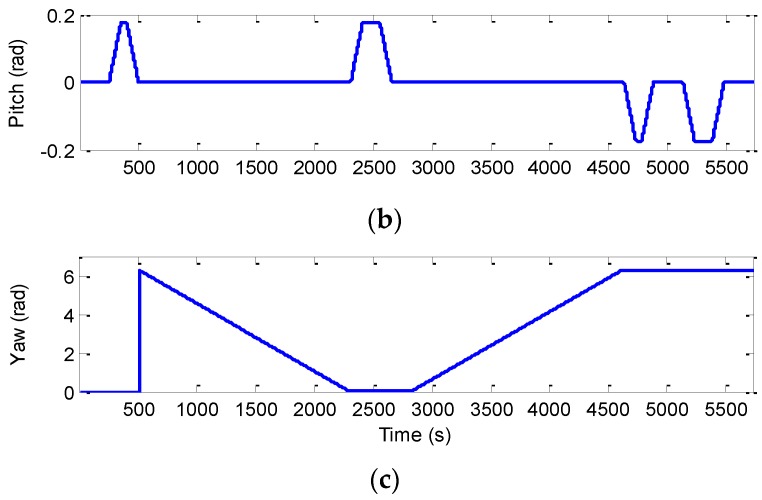
The Euler angles for the case of three-dimensional simulation. (**a**) Roll angle; (**b**) Pitch angle; (**c**) Yaw angle.

The three-dimensional vehicle trajectory for Scenario 2 is shown in Figure 10. Table 6 presents description of the three-dimensional vehicle motion. Figure 11 shows the Euler angles for the case of three-dimensional simulation. The trajectory for this example can be mainly divided into nine time intervals according to the vehicle dynamic characteristics. The vehicle was simulated to conduct constant acceleration and level flight from 0 to 250 s, counter-clockwise turns from 501 to 2310 s and clockwise circular motion from 2821–4630 s. In the three time intervals, highly dynamic maneuvering is involved. The constant-velocity straight-line flight is involved in all the other segments, where the low dynamic motion is considered.

The INS equations describing the three-dimensional inertial navigation state are:

(45) $${\dot{V}}_{e}^{n}=C_{b}^{n}f^{n}-[\Omega (\omega_{en}^{n})+2\Omega (\omega_{ie}^{n})]V_{e}^{n}+g_{l}^{n}$$

The error model employed for INS is a terrestrial INS psi angle error model [24]:

(46) $$\delta \dot{R}=-\omega_{en}\times \delta R+\delta V$$

(47) $$\delta \dot{V}=-(\omega_{ie}+\omega_{in})\times \delta V-\psi \times f+\varepsilon_{a}$$

(48) $$\dot{\psi }=-\omega_{in}\times \psi +\varepsilon_{g}$$

where δR is the position error vector, δV is the velocity error vector, ψ is the attitude error vector, $\omega_{en}$ refers to the rotation rate of the local geographic frame relative to the earth frame, $\omega_{ie}$ refers to the earth rate vector, $\omega_{in}$ refers to the rotation rate of the local geographic frame with respect to the inertial frame, f is the specific force vector, $\varepsilon_{a}$ is the accelerometer error vector and $\varepsilon_{g}$ is the gyro drift rate vector. The state vector include 17 states: three inertial error states each in position, velocity, attitude, accelerometer bias, and gyro bias, and one state each for receiver clock bias and drift:

(49) $$x_{k}={\left[x,y,z,\dot{x},\dot{y},\dot{z},\psi_{x},\psi_{y},\psi_{z},a_{x},a_{y},a_{z},g_{x},g_{y},g_{z},b,d\right]}^{T}$$

A tightly-coupled GPS/INS integration configuration is used. The states and the measurements are related nonlinearly. The nonlinear pseudorange equation can be linearized by expanding Taylor’s series around the approximate (or nominal) user position and neglecting the higher terms. If only the pseudorange observables are available, the linearized measurement equation based on n observables can be written as given by:

(50) $$\left[\begin{array}{c} \rho_{1}\\ \rho_{2}\\ \vdots \\ \rho_{n} \end{array}\right]=\left[\begin{array}{c} {\hat{\rho }}_{1}\\ {\hat{\rho }}_{2}\\ \vdots \\ {\hat{\rho }}_{n} \end{array}\right]+\left[\begin{array}{cccccc} h_{x}^{\left(1\right)} & h_{y}^{\left(1\right)} & h_{z}^{\left(1\right)} & \cdots & 1 & 0\\ h_{x}^{\left(2\right)} & h_{y}^{\left(2\right)} & h_{z}^{\left(2\right)} & \cdots & 1 & 0\\ \vdots & \vdots & \vdots & \cdots & \vdots & \vdots \\ h_{x}^{\left(n\right)} & h_{y}^{\left(n\right)} & h_{z}^{\left(n\right)} & \cdots & 1 & 0 \end{array}\right]x_{k}+\left[\begin{array}{c} v_{\rho_{1}}\\ v_{\rho_{2}}\\ \vdots \\ v_{\rho_{n}} \end{array}\right]$$

where $x_{k}$ is shown as in Equation (49) and $v_{\rho }$ is the measurement noise.

**Figure 12:**
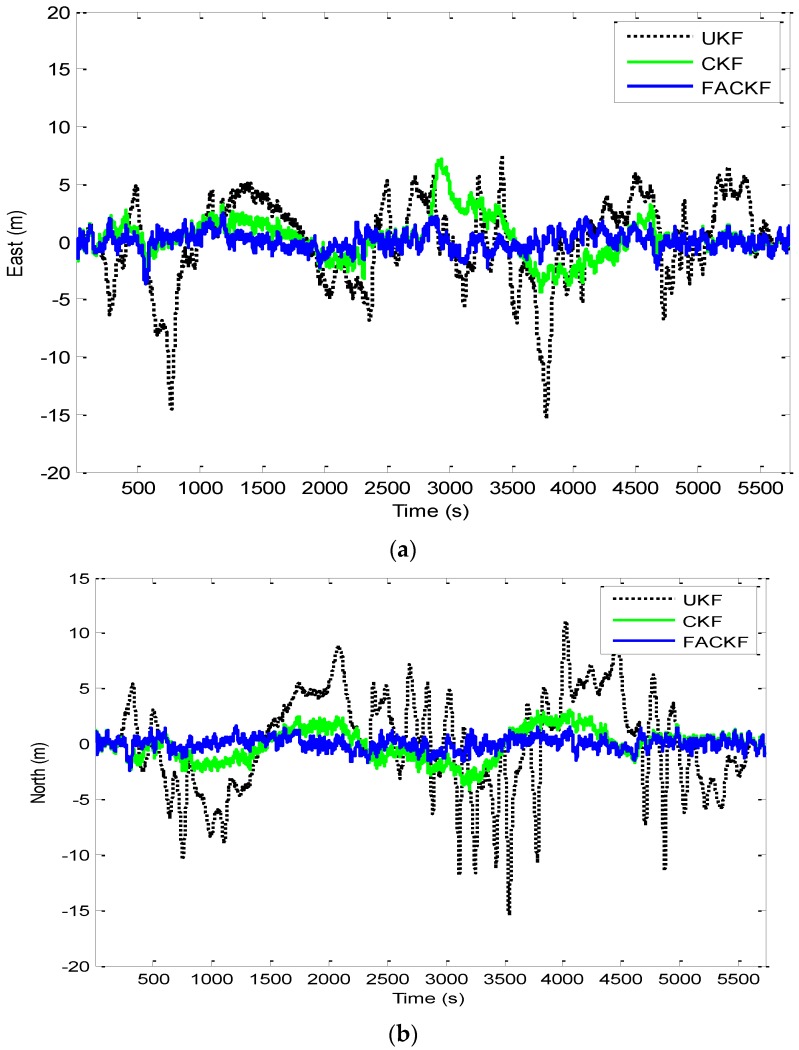

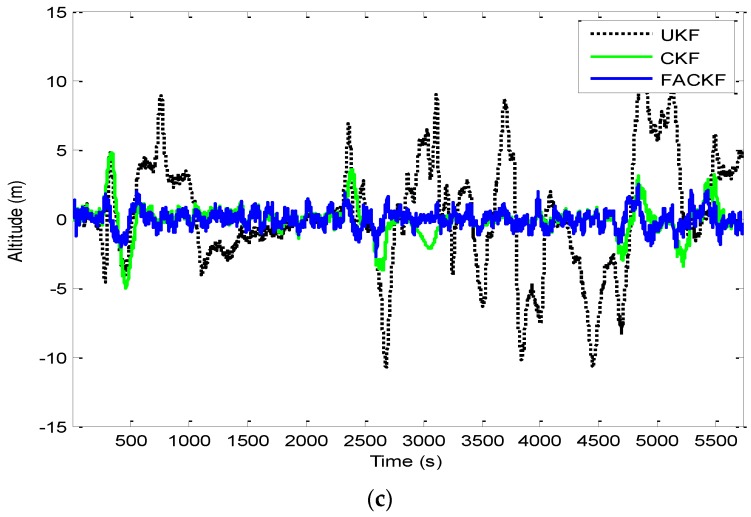
Position errors for UKF, CKF, and FACKF—Scenario 2. (**a**) East position error; (**b**) North position error; (**c**) Altitude error.

**Figure 13:**
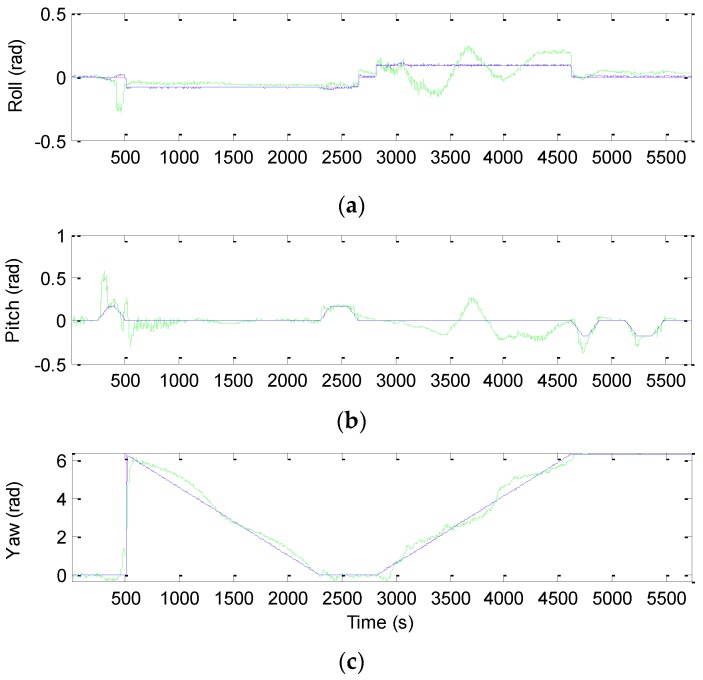
Comparison of Euler angle estimation results: UKF (in green) versus CKF (in blue)—Scenario 2. (**a**) Roll angle; (**b**) Pitch angle; (**c**) Yaw angle.

**Figure 14:**
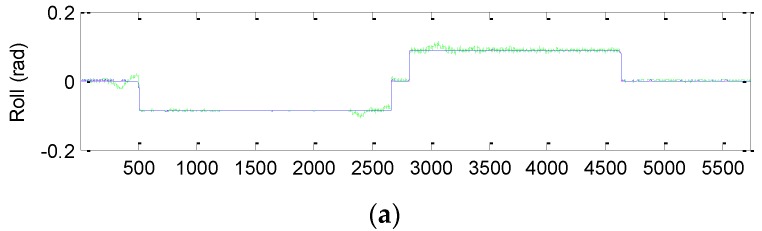

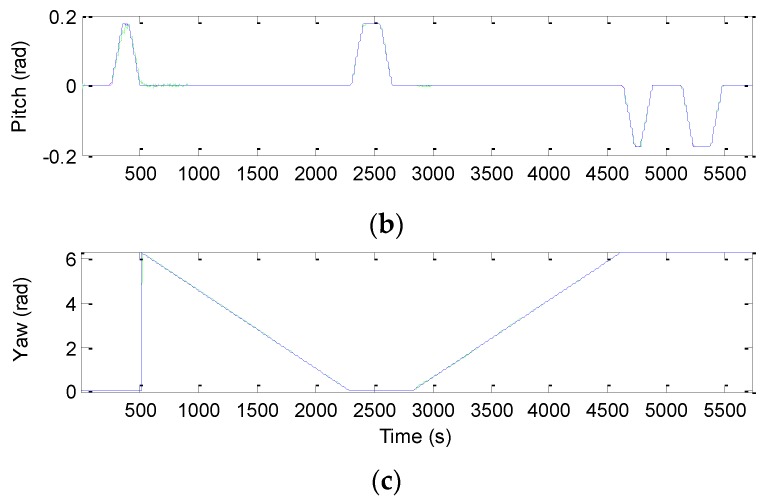
Comparison of Euler angle estimation results: CKF (in green) versus FACKF (in blue)—Scenario 2. (**a**) Roll angle; (**b**) Pitch angle; (**c**) Yaw angle.

**Figure 15:**
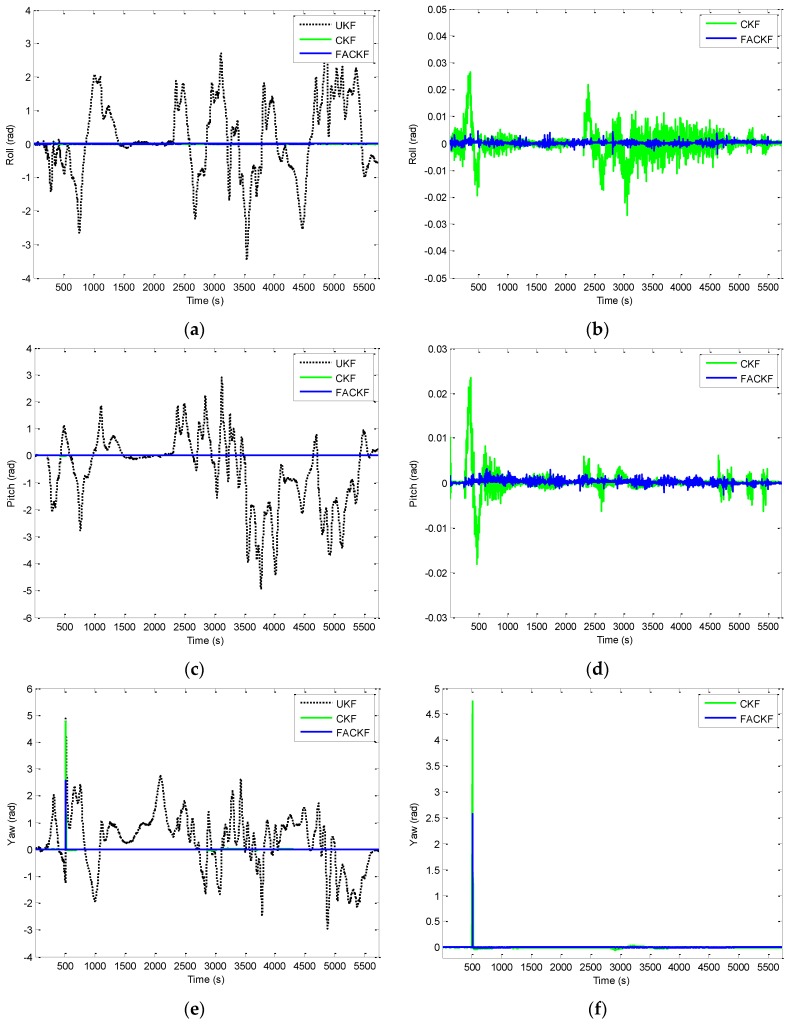
Comparison of Euler angle errors for UKF, CKF, and FACKF—Scenario 2: (**a,c,e**) UKF, CKF and FACKF; and (**b,d,f**) CKF versus FACKF.

The effectiveness of the proposed method for Scenario 2 is given by the results shown from Figure 12, Figure 13, Figure 14 and Figure 15. Figure 12 provides the position errors based on the three nonlinear filter approaches: UKF, CKF and FACKF. It can be seen that the state estimate of the conventional CKF has deviated from the true state in high dynamic segments, while the FACKF provides remarkable improvement. Comparison of Euler angle estimation results for UKF versus CKF is shown in Figure 13; and comparison of Euler angle estimation results for CKF versus FACKF is shown in Figure 14. The estimation results of Euler angles based on the UKF are unreliable, while the CKF is able to provide acceptable results. When using the FACKF, the results are further improved. The reason is that the FACKF involves the use of appropriate weighting factor online whereas the other three approaches do not offer this flexibility and depend mainly on the fixed parameters of process noise covariance matrix based on the prior knowledge. Figure 15 presents the comparison of Euler angle errors for UKF, CKF, and FACKF, in which the results for the three approaches are shown as in the three plots on the left column and, for better readability, CKF versus FACKF on the right column. Table 7 provides the summary of RMS errors and the time consumption for the 3D navigation case. Comparison of RMS errors is illustrated in Figure 11, which demonstrates that FACKF outperforms significantly the other approaches: EKF, UKF, and CKF.

Based on the results, several important remarks are given as follows:

(1) During the time intervals the vehicle is conducting maneuvering, i.e., circular motion, turn motion, constant acceleration, or variable acceleration, the model mismatch to the actual situation leads to increased errors, as can be seen from the solution obtained by the conventional EKF. The CKF is about to converge in the high dynamic regions where there still exist noticeably large errors for the UKF-based solutions.
(2) Since the use of fixed value of covariance matrix cannot reflect the realistic vehicle dynamics, the performance of the nonlinear filters degrades due to such uncertainties on parameter values. To improve the performance of the CKF, a robust technique that the FLAS mechanism is incorporated for dynamically tuning the process noise covariance in the CKF framework. Remarkable improvement in estimation accuracy is obtained using the FACKF algorithm.
(3) For the three-dimensional case, the estimation results of Euler angles based on the UKF are unreliable, while the CKF is able to provide acceptable results. When using the FACKF, the results are further improved.
(4) The results from the two illustrative examples demonstrate that, by monitoring the innovation information based on DOD parameters, the FACKF possesses superior capability to detect the change in vehicle dynamics and adjust the scaling factor so as to prevent the divergence and remain better navigation accuracy. For the segments with sharp turns or abrupt maneuvers involved, the performance improvement becomes obvious.
(5) The results show that through tuning of the process noise covariance, the FLAS helps to resolve the problem on noise uncertainty in the CKF for fusing the integrated navigation system data. Therefore, when a designer does not have sufficient information to develop the precise model, the proposed approach provides a useful alternative for designing the GPS/INS integration.

## 5. Conclusions

This paper has presented a sensor fusion method for the GPS/INS integrated navigation systems. The proposed approach is based on the combination of cubature Kalman filter (CKF) to treat the system nonlinearity, and fuzzy logic adaptive system (FLAS) to tune the covariance matrix of the process noise during the vehicle maneuvering.

The proposed FLAS-assisted CKF enhances the robustness of CKF with better treatment to system models including statistical uncertainties (mainly for adjusting the covariance matrix of the process noise) through the FLAS. If the nonlinear filter does not perform satisfactorily well, the FLAS would provide an appropriate weighting factor to improve the navigation accuracy of the CKF. The FLAS adaptive mechanism provides adequate tuning of the weighting factor, which enables the system to achieve a balance between estimation accuracy and robustness. Based on the T-S fuzzy model, the proposed FACKF scheme timely performs effective detection of vehicle maneuvering motion and exhibits robustness against the divergence problem. The FACKF method is designed to overcome the possible degradation problems caused by modeling errors on noise uncertainty, so as to improve the navigation accuracy in highly dynamic segments without sacrificing precision in the other regions.

To validate the effectiveness of the proposed method, two illustrative examples for the GPS/INS integration have been presented, one for the two-dimensional land vehicle navigation using the loosely-coupled configuration; the other for the three-dimensional navigation using the tightly-coupled configuration. Evaluation of navigation performance among various nonlinear filters, including EKF, UKF, CKF, and FACKF, has been presented. As an enhanced version of CKF, the FACKF possesses superior performance improvement in navigational accuracy and reveals very good potential as an alternative navigation state estimator for the GPS/INS navigation design.

## Figures and Tables

**Table 1:** Implementation algorithm for the unscented Kalman filter.

- Initialization: Initialize state vector ${\hat{x}}_{0|0}$ and state covariance matrix $P_{0|0}$
- Time update
(1) The transformed set is given by instantiating each point through the process model
(9) $$\zeta_{i,k|k-1}=f(X_{i,k-1}),\;i=0,\ldots ,2n$$
(2) Predicted mean
(10) $${\hat{x}}_{k|k-1}=\sum_{i=0}^{2n}W_{i}^{\left(m\right)}\zeta_{i,k|k-1}$$
(3) Predicted covariance
(11) $$P_{k|k-1}=\sum_{i=0}^{2n}W_{i}^{\left(c\right)}[\zeta_{i,k|k-1}-{\hat{x}}_{k|k-1}]{\left[\zeta_{i,k|k-1}-{\hat{x}}_{k|k-1}\right]}^{T}+Q_{k-1}$$
(4) Instantiate each of the prediction points through observation model
(12) $$Z_{i,k|k-1}=h(\zeta_{i,k|k-1})$$
(5) Predicted observation
(13) $${\hat{z}}_{k|k-1}=\sum_{i=0}^{2n}W_{i}^{\left(m\right)}Z_{i,k|k-1}$$
-Measurement update
(6) Innovation covariance
(14) $$P_{\text{zz}}=\sum_{i=0}^{2n}W_{i}^{\left(c\right)}[Z_{i,k|k-1}-{\hat{z}}_{k|k-1}]{\left[Z_{i,k|k-1}-{\hat{z}}_{k|k-1}\right]}^{T}+R_{k}$$
(7) Cross covariance
(15) $$P_{xz}=\sum_{i=0}^{2n}W_{i}^{\left(c\right)}[\zeta_{i,k|k-1}-{\hat{x}}_{k|k-1}]{\left[Z_{i,k|k-1}-{\hat{z}}_{k|k-1}\right]}^{T}$$
(8) Performing update
(16) $$K_{k}=P_{\text{xz}}P_{\text{zz}}^{-1}$$
(17) $${\hat{x}}_{k|k}={\hat{x}}_{k|k-1}+K_{k}(z_{k}-{\hat{z}}_{k|k-1})$$
(18) $$P_{k|k}=P_{k|k-1}-K_{k}P_{\text{zz}}K_{k}^{T}$$

**Table 2:** Implementation algorithm for the cubature Kalman filter.

- Initialization: Initialize state vector ${\hat{x}}_{0|0}$ and state covariance matrix $P_{0|0}$
- Time update
(1) Factorize the covariance
(22) $$P_{k-1|k-1}=S_{k-1|k-1}S_{k-1|k-1}^{T}$$
(2) Evaluate the cubature points
(23) $$X_{i,k-1|k-1}=S_{k-1|k-1}\xi_{i}+{\hat{x}}_{k-1|k-1}$$
(3) Evaluate the propagated cubature points through the process model
(24) $$X_{i,k|k-1}^{*}=f(X_{i,k-1|k-1})$$
(4) Estimate the predicted mean
(25) $${\hat{x}}_{k|k-1}=\sum_{i=1}^{2n}\omega_{i}X_{i,k|k-1}^{*}$$
(5) Estimate the predicted error covariance
(26) $$P_{k|k-1}=\sum_{i=1}^{2n}\omega_{i}X_{i,k|k-1}^{*}X_{i,k|k-1}^{{*}^{T}}-{\hat{x}}_{k|k-1}{\hat{x}}_{k|k-1}^{T}+Q_{k-1}$$
- Measurement update
(6) Factorize the covariance
(27) $$P_{k|k-1}=S_{k|k-1}S_{k|k-1}^{T}$$
(7) Evaluate the cubature points
(28) $$X_{i,k|k-1}=S_{k|k-1}\xi_{i}+{\hat{x}}_{k|k-1}$$
(8) Evaluate the propagated cubature points through observation model
(29) $$Z_{i,k|k-1}=h(X_{i,k|k-1})$$
(9) Evaluate the propagated observation
(30) $${\hat{z}}_{k|k-1}=\sum_{i=1}^{2n}\omega_{i}Z_{i,k|k-1}$$
(10) Estimate the innovation covariance
(31) $$P_{zz}=\sum_{i=1}^{2n}\omega_{i}Z_{i,k|k-1}Z_{i,k|k-1}^{T}-{\hat{z}}_{k|k-1}{\hat{z}}_{k|k-1}^{T}+R_{k}$$
(11) Estimate the cross-covariance
(32) $$P_{xz}=\sum_{i=1}^{2n}\omega_{i}X_{i,k|k-1}Z_{i,k|k-1}^{T}-{\hat{x}}_{k|k-1}{\hat{z}}_{k|k-1}^{T}$$
(12) Perform update state vector ${\hat{x}}_{k|k}$ and its covariance matrix $P_{k|k}$
(33) $$K_{k}=P_{xz}P_{zz}^{-1}$$
(34) $${\hat{x}}_{k|k}={\hat{x}}_{k|k-1}+K_{k}(z_{k}-{\hat{z}}_{k|k-1})$$
(35) $$P_{k|k}=P_{k|k-1}-K_{k}P_{zz}K_{k}^{T}$$

**Table 3:** Description of the vehicle motion for Scenario 1.

Segment Number	Time Interval (s)	Motion
1	(0–300)	Constant velocity straight line
2	(301–500)	Counter-clockwise circular motion
3	(501–600)	Constant velocity straight line
4	(601–700)	Counter-clockwise turn
5	(701–1000)	Constant velocity straight line
6	(1001–1100)	Counter-clockwise turn
7	(1101–1400)	Constant velocity straight line
8	(1401–1500)	Counter-clockwise turn
9	(1501–1600)	Constant velocity straight line
10	(1601–1700)	Clockwise turn
11	(1701–1800)	Constant velocity straight line
12	(1801–1900)	Clockwise turn
13	(1901–2000)	Constant velocity straight line

**Table 4:** Summary of RMS errors and the time consumption for Scenario 1.

	RMS Errors (in Units of *m* for Positions and Rad for Angles, Respectively)			Time Consumption (s)
	East	North	Yaw	
EKF	10.2400	8.8917	0.0511	6.144
UKF	2.3966	3.1636	0.0236	12.702
CKF	1.4524	1.6161	0.0165	11.872
FACKF	0.3634	0.2312	0.0096	13.162

**Table 5:** INS error specifications (from Crista IMU Specifications [23]).

	Gyros	Accelerometers
Range	±300°/s	±10 G
Scale Factor Error	<1% (@ 25 °C)	<1% (@ 25 °C)
	(i.e., < 3°/s)	(i.e., < 100 mG or 0.98 m/s^2^)
In-Run Bias Error		
Fixed temperature	<0.2°/s (warmed up)	<25 mG (0.245 m/s^2^)
Over temperature	<0.6°/s	<51 mG (0.500 m/s^2^)
Noise (1σ, no oversamples)	<± 0.7°/s	<±12 mG (0.120 m/s^2^)

**Table 6:** Description of the vehicle motion for Scenario 2.

Segment Number	Time Interval (s)	Motion
1	(0–250)	Constant acceleration level flight
2	(251–500)	Climbing
3	(501–2310)	Counter-clockwise circular motion
4	(2311–2820)	Climbing
5	(2821–4630)	Clockwise circular motion
6	(4631–4880)	Descending
7	(4881–5120)	Constant velocity level flight
8	(5121–5470)	Descending
9	(5471–5740)	Constant velocity level flight

**Table 7:** Summary of RMS errors and the time consumption for Scenario 2.

RMS Errors (in Units of m for Positions and Rad for Angles, Respectively)							Time Consumption (s)
	East	North	Altitude	Roll	Pitch	Yaw	
EKF	9.0112	7.6160	6.3685	5.1543	9.2497	6.7151	32.287
UKF	3.7776	4.4502	4.0834	1.1380	1.4199	1.0941	38.418
CKF	1.7921	1.4048	1.1736	0.0047	0.0027	0.0965	37.742
FACKF	0.8075	0.5933	0.6698	0.0005	0.0004	0.0346	40.106
